# A multi-center study to evaluate the impact of germline BRCA1 and BRCA2 mutations on ovarian cancer survival

**DOI:** 10.1186/1897-4287-10-S2-A27

**Published:** 2012-04-12

**Authors:** KL Bolton, G Chenevix-Trench, C Goh, S Sadetzki, SJ Ramus, SA Gayther, SJ Chanock, AC Antoniou, PDP Pharoah

**Affiliations:** 1 Division of Cancer Epidemiology and Genetics, National Cancer Institute, Bethesda MD 20892, USA; 2 Queensland Institute of Medical Research, Locked Bag 2000, Royal Brisbane Hospital, Herston, QLD 4029, Australia; 3 Addenbrooke’s Hospital, Hills Road, Cambridge CB2 OQQ, UK; 4 Cancer and Radiation Epidemiology Unit, Gertner Institute for Epidemiology and Health Policy, Sheba Medical Center, Tel Hashomer, 52621, Israel; 5 Sackler Faculty of Medicine, Tel-Aviv University, Tel-Aviv, Israel; 6 Gynaecological Cancer Research Laboratories, UCL EGA Institute for Women's Health, University College London, London, UK; 7 Department of Preventive Medicine, Keck School of Medicine University of Southern California, Los Angeles, California, 90033, USA; 8 Centre for Cancer Genetic Epidemiology, Department of Public Health and Primary Care, University of Cambridge, Strangeways Research Laboratory, Worts Causeway, Cambridge CB1 8RN, UK; 9 Cancer Research United Kingdom Department of Oncology and Department of Public Health and Primary Care, University of Cambridge, Strangeways Research Laboratory, Worts Causeway, Cambridge CB1 8RN, UK

## Background

Approximately 10 percent of women with invasive epithelial ovarian cancer (EOC) carry deleterious germline mutations in *BRCA1* or *BRCA2*. However, the impact of these mutations on ovarian cancer prognosis remains unclear.

## Methods

We performed an international multi-center study of 1,470 EOC cases with pathogenic germline mutations in *BRCA1* (1,134) or *BRCA2* (336) and 2,814 non-carriers. Our goal was to further characterize the survival of *BRCA* carriers with EOC compared to non-carriers and to determine whether *BRCA1* and *BRCA2* carriers show similar survival patterns. Cox proportional hazards regression, both unadjusted and adjusted for other prognostic variables, was used to measure differences in overall survival during the five years following diagnosis.

## Results

The five-year overall survival was 36 percent for non-carriers, 44 percent for *BRCA1* carriers and 52 percent for *BRCA2* carriers. After adjusting for study and year of diagnosis, *BRCA1* and BRCA2 carriers showed a more favorable survival than non-carriers (*BRCA1*, HR=0.78; 95% CI=0.68-0.89, P=2x10^-4^; *BRCA2*, HR = 0.61; 95% CI=0.50-0.76, P=6x10^-6^;). These survival differences remained after adjustment for stage, grade, histology and age at diagnosis (*BRCA1*, HR=0.73, 95% CI=0.64-0.84, P=2x10^-5^; *BRCA2*, HR = 0.49, 95% CI=0.39-0.61, P=3x10^-10^).

## Conclusions

We observed a significantly improved survival in germline *BRCA1* and *BRCA2* mutation carriers with EOC compared to non-carriers. *BRCA2* carriers had the most favorable outcome with a distinct clinical course from *BRCA1* carriers. The magnitude of the differences we observed highlight the need for clinical trials in EOC to be stratified by *BRCA1/2* status and suggest that the routine testing of women presenting with high-grade serous EOC may be warranted.

